# Hydrostatic Pressure Influences HIF-2 Alpha Expression in Chondrocytes

**DOI:** 10.3390/ijms16011043

**Published:** 2015-01-05

**Authors:** Hiroaki Inoue, Yuji Arai, Tsunao Kishida, Ryu Terauchi, Kuniaki Honjo, Shuji Nakagawa, Shinji Tsuchida, Tomohiro Matsuki, Keiichirou Ueshima, Hiroyoshi Fujiwara, Osam Mazda, Toshikazu Kubo

**Affiliations:** 1 Department of Orthopaedics, Graduate School of Medical Science, Kyoto Prefectural University of Medicine, Kawaramachi-Hirokoji, Kamigyo-ku, Kyoto 602-8566, Japan; E-Mails: hinoue@koto.kpu-m.ac.jp (H.I.); ryutel@mbox.kyoto-inet.or.jp (R.T.); kuniakih@koto.kpu-m.ac.jp (K.H.); shushi@koto.kpu-m.ac.jp (S.N.); tuchi-kf@koto.kpu-m.ac.jp (S.T.); tmatsuki@koto.kpu-m.ac.jp (T.M.); ueshima@koto.kpu-m.ac.jp (K.U.); fjwr@koto.kpu-m.ac.jp (H.F.); tkubo@koto.kpu-m.ac.jp (T.K.); 2 Department of Immunology, Graduate School of Medical Science, Kyoto Prefectural University of Medicine, Kawaramachi-Hirokoji, Kamigyo-ku, Kyoto 602-8566, Japan; E-Mails: tsunao@koto.kpu-m.ac.jp (T.K.); mazda@koto.kpu-m.ac.jp (O.M.)

**Keywords:** hypoxia inducible factor (HIF)-2α, hydrostatic pressure, chondrocytes, hypertrophic differentiation, osteoarthritis, cartilage degeneration, inflammation

## Abstract

Hypoxia-inducible factor (HIF)-2α is considered to play a major role in the progression of osteoarthritis. Recently, it was reported that pressure amplitude influences HIF-2α expression in murine endothelial cells. We examined whether hydrostatic pressure is involved in expression of HIF-2α in articular chondrocytes. Chondrocytes were cultured and stimulated by inflammation or hydrostatic pressure of 0, 5, 10, or 50 MPa. After stimulation, heat shock protein (HSP) 70, HIF-2α, nuclear factor kappa B (NF-κB), matrix metalloproteinase (MMP)-13, MMP-3, and vascular endothelial growth factor (VEGF) gene expression were evaluated. The levels of all gene expression were increased by inflammatory stress. When chondrocytes were exposed to a hydrostatic pressure of 5 MPa, HIF-2α, MMP-13, and MMP-3 gene expression increased significantly although those of HSP70 and NF-κB were not significantly different from the control group. In contrast, HIF-2α gene expression did not increase under a hydrostatic pressure of 50 MPa although HSP70 and NF-κB expression increased significantly compared to control. We considered that hydrostatic pressure of 5 MPa could regulate HIF-2α independent of NF-κB, because the level of HIF-2α gene expression increased significantly without upregulation of NF-κB expression at 5 MPa. Hydrostatic pressure may influence cartilage degeneration, inducing MMP-13 and MMP-3 expression through HIF-2α.

## 1. Introduction

Osteoarthritis (OA) is a degenerative disease of the articular cartilage, impairing activity of daily living and quality of life due to arthralgia, limitation of the range of joint motion, and joint swelling. Mechanical loading is considered to be strongly involved in degeneration of articular cartilage. Takahashi *et al.* [1] showed that continuous hydrostatic pressure inhibited proteoglycan synthesis in a human chondrocyte-like cell line and Nakamura *et al.* [2] showed that induced apoptosis depending on pressure amplitude and/or duration in chondrocytes cultured on alginate beads. Therefore, excessive mechanical stress induces degeneration and destruction of articular cartilage, causing OA.

Hypertrophic differentiation characterized by secretion of type 10 collagen is involved in the initiation of OA. Hypertrophic differentiated chondrocytes product matrix metalloproteinase (MMP)s, resulting in progression of cartilage degeneration. Hypoxia-inducible factor (HIF)-2α is a transcription factor that strongly induces type 10 collagen in chondrocytes [3,4]. Moreover, HIF-2α induces expression of a wide range of factors involved in endochondral ossification, such as MMPs, vascular endothelial growth factor (VEGF), and Indian hedgehog signals, in articular chondrocytes. Recently, it was reported that pressure amplitude influences HIF-2α expression in murine endothelial cells [5,6]. However, the influence of pressure on cartilage tissue is unknown. The present study was performed to analyze the influence of hydrostatic pressure on hypertrophic differentiation-related gene expression in cultured chondrocytes.

## 2. Results

We first examined the appropriateness of culture environments and primers by stimulating inflammation stress to cultured chondrocytes. Then, hydrostatic pressure was exposed to primary cultured chondrocytes measuring gene expression related to hypertrophic differentiation.

### 2.1. Influence of Inflammation to Chondrocytes

**Figure 1:**
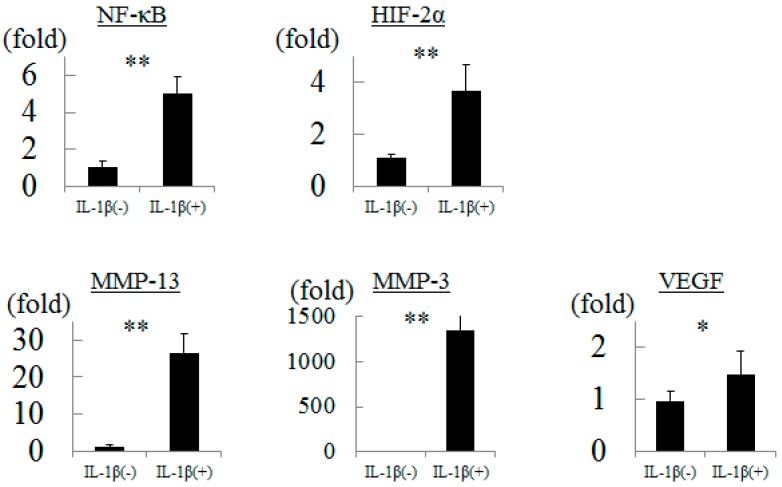
Gene expressions in chondrocytes after interleukin-1β stimulation. * *p* < 0.05, ** *p* < 0.01.

We measured gene expression in cultured chondrocytes after treated with interleukin (IL)-1β. The levels of NF-κB, HIF-2α, MMP-13, MMP-3, and VEGF gene expression were significantly increased by 5.0-, 3.7-, 26.5-, 1356-, and 1.5-fold, respectively, by inflammatory stress (Figure 1).

### 2.2. Influence of Hydrostatic Pressure to Chondrocytes

**Figure 2:**
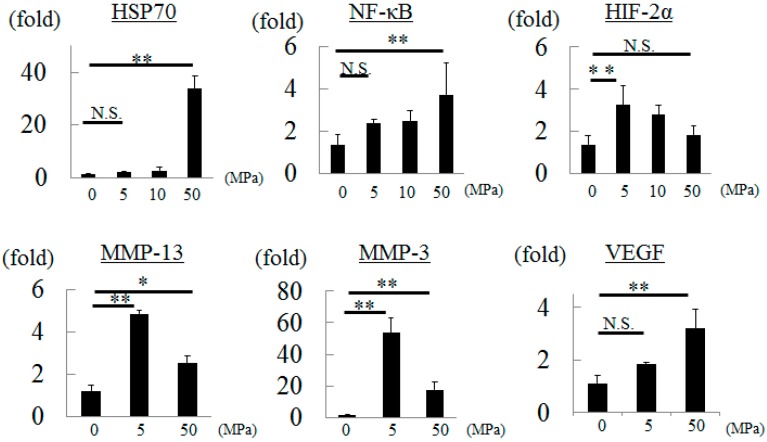
Gene expressions in chondrocytes after hydrostatic pressurization. * *p* < 0.05, ** *p* < 0.01, and N.S.: no significant.

When chondrocytes were exposed to a hydrostatic pressure of 5 MPa, HIF-2α, MMP-13, and MMP-3 gene expression were significantly increased by 2.4-, 4.0-, and 40-fold, respectively. However, those of HSP70 and NF-κB were not significantly different compared to the control group (Figure 2).

In contrast, HIF-2α gene expression did not increase although those of HSP70 and NF-κB increased significantly compared to the control group under a hydrostatic pressure of 50 MPa. MMP-13 and MMP-3 gene expression in chondrocytes exposed to 50 MPa increased significantly compared to controls, but the rate of increase was low compared to the 5 MPa group. The level of VEGF gene expression in the 50 MPa group was significantly increased compared to the control group (Figure 2).

## 3. Discussion

Inflammatory stimulation enhances NF-κB, HIF-2α, MMP-13, MMP-3, and VEGF gene expression in cultured chondrocytes [5]. In this study, we confirmed the appropriateness of culture environments and primers because the levels of expression of all of these genes increased as described in previous reports. HIF-2α is not produced in normal articular cartilage, but is produced in human OA cartilage. It has also been clarified that surgical destabilization of the medial meniscus model using HIF-2α knockout mice showed suppression of OA progression and inhibition of MMP-13 gene expression in addition to HIF-2α compared to wild-type mice [3]. Therefore, HIF-2α has attracted a great deal of attention as a treatment target for OA at the molecular level. Meanwhile, as it has been reported that HIF-2α is upregulated by prolonged mechanical stretching in rat inferior vena cava [6] and HIF-2α-deficient embryonic vascular endothelial cells show downregulation of integrin [7], the expression of HIF-2α may be associated with pressure or its sensor. However, there have been no previous reports regarding the relation between HIF-2α and pressure in cartilage tissue. Therefore, the present study was performed to determine the relation between HIF-2α and hydrostatic pressure.

Previously, we reported that continuous hydrostatic pressure induced expression of the stress protein, HSP70, in a pressure-dependent manner in a study using a chondrocyte-like cell line [1]. In this study, HSP70 gene expression did not change with exposure to a hydrostatic pressure of 5 or 10 MPa, but showed a significant increase on exposure to 50 MPa. Therefore, we performed the following experiments considering 50 MPa as excessive hydrostatic pressure.

HSP70 has been reported to mediate NF-κB expression in hepatocarcinoma cells [8]. Compared to the control group, there were no significant increase in HSP70 and NF-κB gene expression at 5 MPa, but expression of both increased significantly at 50 MPa in the present study. Thus, we speculate that HSP70 induced by hydrostatic pressure may have a role in the expression of NF-κB. With regard to the relationship between NF-κB and HIF-2α, Saito *et al.* [3] demonstrated that NF-κB is a potent inducer of HIF-2α in HIF-2α promoter assay. However, we considered the possibility that a hydrostatic pressure of 5 MPa could actually regulate HIF-2α independent of NF-κB, because HIF-2α gene expression increased significantly without upregulation of NF-κB expression at a hydrostatic pressure of 5 MPa in the present study. Further experiments are needed to prove this. The differences between the results of the present study and those of previous reports may have been because we analyzed the influence of hydrostatic pressure alone in cultured chondrocytes. In addition, these discrepancies may also be explained by the influence of other factors produced by activation of HIF-2α that may act as negative feedback, such as HIF-3α [9]. MMP-13 and MMP-3 were shown to be induced by HIF-2α [7,10]. The levels of MMP-13 and MMP-3 gene expression also increased most at 5 MPa compared to the controls in the present study. These observations indicated that hydrostatic pressure influences cartilage degeneration, inducing MMP-13 and MMP-3 expression through HIF-2α. Although it has been reported that VEGF is induced by HIF-2α, explaining the mechanism of regulation of VEGF is difficult because VEGF is induced by bone morphogenetic protein-2, Rho, and HIF-1α [11,12]. The inconsistency between expression of HIF-2α and that of VEGF may be due to the influence of factors other than HIF-2α.

## 4. Experimental Section

This study was conducted in accordance with the animal research guidelines of Kyoto Prefectural University of Medicine, Kyoto, Japan, and accepted by animal experiment committee as No. 24-3.

### 4.1. Preparation of Chondrocytes

Male Japanese white rabbits (Shimizu Laboratory Supplies, Kyoto, Japan) weighing 2 kg were killed by a lethal dose of sodium pentobarbital (Nembutal; Abbott, Abbott Park, IL, USA), and the cartilage was collected aseptically from the bilateral knee, hip, and shoulder joints. The specimens were minced into small pieces and treated with 0.015% trypsin (Gibco BRL, Gaithersburg, MD, USA) for 1 h, followed by treatment with 0.025% collagenase (Collagenase L; Nitta Gelatin, Osaka, Japan) for 8 hours. Isolated chondrocytes were cultured for 1 week at 37 °C in 5% CO_2_ 95% humidified air (standard conditions) in Dulbecco’s modified Eagle’s medium (DMEM) containing 4.5 gm/L glucose, 0.584 gm/L l-glutamine, and 0.11 gm/L sodium pyruvate (Nacalai Tesque, Kyoto, Japan), which was supplemented with 10% fetal bovine serum (FBS) (Trace Biosciences, Melbourne, Victoria, Australia), 100 units/mL penicillin, and 100 µg/mL streptomycin (Gibco BRL).

### 4.2. IL-1β Treatment

IL-1β stimulation enhances nuclear factor-kappa B (NF-κB), HIF-2α, MMP-13, MMP-3, and vascular endothelial growth factor (VEGF) gene expression in rabbit chondrocytes. In this study, we conducted a similar experiment to examine expression of these genes as positive controls. Chondrocytes were maintained as a monolayer, and on day 3 of culture the cells were treated with 4 ng/mL of IL-1β (R&D systems, Minneapolis, MN, USA) for 24 h.

### 4.3. Pressure Application

The cells reached confluence following approximately 1 week of culture, and were then exposed to a hydrostatic pressure of 5–50 MPa. Petri dishes were placed in a deformable polytetrafluoroethylene (Teflon) pouch that was filled with serum-free DMEM containing the same concentrations of kanamycin and l-glutamine as mentioned above and packed completely after air bubbles were removed. The pouch was then placed in a stainless steel pressurization vessel (inside diameter 65 × 90 mm), equipped with an oil pressure apparatus (type KP5B: Hikari Koatsu, Hiroshima, Japan). Kerosene was used as the pressure medium, and pressure was transmitted to the cells through the packed Teflon pouch and then the culture medium in a gas-free environment. The temperature was maintained at 37 °C by use of a thermostat located in the pressurization vessel. We previously confirmed that there were no changes in the pH of the culture medium prior to and following the exposure to hydrostatic pressure. The Teflon pouch was then exposed to a hydrostatic pressure of 5, 10, or 50 MPa for 2 h. After depressurization, 3 mL of fresh serum-free DMEM was added to each Petri dish and kept at atmospheric pressure in the 5% CO_2_ environment until analysis. Cells seeded in Petri dishes and placed in the same apparatus under the same conditions were used as non-pressurized controls.

### 4.4. Quantitative Real-Time Reverse Transcription-Polymerase Chain Reaction

We studied change of HIF-2α expression under excessive stress. HSP70 expression was measured as index of excessive stress. Takahashi *et al*. [1] have been reported gene expression of HSP70 elevation was observed at 30 min following the release of 50 MPa pressure using chondrocyte-like cell line. In contrast, the elevation level was decreased at 4 h. Thirty minutes after depressurization or 24 h after IL-1β stimulation, total RNA was extracted using Sepasol-RNA super II (Nacalai Tesque) and subjected to quantitative real-time reverse transcription-polymerase chain reaction (RT-PCR) using a Biosystems 7300 (Applied Biosystems, Foster City, CA, USA). RT-PCR was performed for HIF-2α, for NF-κB, for MMP-3, for MMP-13, and for VEGF with gene-specific primers and TaqMan probe (Table 1). Moreover, HSP70 gene expression was used as a marker for evaluation of hydrostatic pressure exposure of cultured chondrocytes. Quantitative RT-PCR was performed using a Biosystems 7300 with TaqMan Assay-on Demand gene expression primer/probe sets (Applied Biosystems) for HSP70 (Assay ID; AJ297379). To quantify changes in gene expression, the comparative Ct method was used to calculate the relative fold changes normalized relative to ribosomal RNA. Results are shown as the means of three samples, with each sample assayed in duplicate.

**Table 1:** Primers used for real-time RT-PCR.

Gene	Primer Sequence	Probe No.
HIF-2α	Forward: agcttcctgcggacacac Reverse: cctcggcttcagactcattt	73
NF-κB	Forward: aagaccaggtttccgaggac Reverse: tgcaccatgaggaaagaagtt	133
MMP-3	Forward: tggacctggaaatgttttgg Reverse: atcaaagtgggcatctccat	72
MMP-13	Forward: cctcttcttctccggaaacc Reverse: ggtagtcttggtccatggtatga	50
VEGF	Forward: ctacctccaccatgccaagt Reverse: ccacttcgtggggtttattg	29
18S ribosomal RNA	Forward: atgagtccactttaaatcctttaacga Reverse: ctttaatatacgctattggagctggaa	-

### 4.5. Statistical Analysis

All data are presented as the mean ± standard deviation (SD). The data were analyzed by analysis of variance (ANOVA), and post hoc testing was performed using the Tukey-Kramer test.

## 5. Conclusions

In conclusion, we showed that expression of HIF-2α in cultured chondrocytes is induced independent of NF-κB expression under a hydrostatic pressure of 5 MPa. These results may help in elucidation of the pathology of OA because HIF-2α and MMPs, acting downstream of HIF-2α, are involved in initiation and progression of OA.
